# Gut-dependent inflammation and alterations of the intestinal microbiota in individuals with perinatal HIV exposure and different HIV serostatus

**DOI:** 10.1097/QAD.0000000000003324

**Published:** 2022-07-22

**Authors:** Camilla Tincati, Monica Ficara, Francesca Ferrari, Matteo Augello, Laura Dotta, Claudia Tagliabue, Alfredo Diana, Vittoria Camelli, Lorenzo Iughetti, Raffaele Badolato, Monica Cellini, Giulia Marchetti

**Affiliations:** a Clinic of Infectious Diseases and Tropical Medicine, San Paolo Hospital, ASST Santi Paolo e Carlo, Department of Health Sciences, University of Milan, Milan; b Division of Paediatric Oncology-Haematology, Policlinico Hospital, Modena; c Pediatric Clinic and ‘A. Nocivelli’ Institute for Molecular Medicine, Spedali Civili Hospital, Department of Clinical and Experimental Sciences, University of Brescia, Brescia; d Fondazione IRCCS Ca’ Granda Ospedale Maggiore Policlinico, Milan; e Section of Pediatrics, Department of Translational Medical Science, University Federico II, Naples; f Department of Sciences of Public Health and Pediatrics, University of Turin, Turin; g Pediatric Unit, Azienda Ospedaliero-Universitaria Policlinico, Modena, Italy.

**Keywords:** gut, HIV-exposed infected, HIV-exposed uninfected, inflammation, microbiome

## Abstract

HIV-exposed infected (HEI) and uninfected (HEU) children represent the two possible outcomes of maternal HIV infection. Modifications of the intestinal microbiome have been linked to clinical vulnerability in both settings, yet whether HEI and HEU differ in terms of gut impairment and peripheral inflammation/activation is unknown.

**Design:**

We performed a cross-sectional, pilot study on fecal and plasma microbiome as well as plasma markers of gut damage, microbial translocation, inflammation and immune activation in HIV-infected and uninfected children born from an HIV-infected mother.

**Methods:**

Fecal and plasma microbiome were determined by means of 16S rDNA amplification with subsequent qPCR quantification. Plasma markers were quantified via ELISA.

**Results:**

Forty-seven HEI and 33 HEU children were consecutively enrolled. The two groups displayed differences in fecal beta-diversity and relative abundance, yet similar microbiome profiles in plasma as well as comparable gut damage and microbial translocation. In contrast, monocyte activation (sCD14) and systemic inflammation (IL-6) were significantly higher in HEI than HEU.

**Conclusion:**

In the setting of perinatal HIV infection, enduring immune activation and inflammation do not appear to be linked to alterations within the gut. Given that markers of activation and inflammation are independent predictors of HIV disease progression, future studies are needed to understand the underlying mechanisms of such processes and elaborate adjuvant therapies to reduce the clinical risk in individuals with perinatal HIV infection.

## Introduction

Maternal HIV infection has two possible and opposite outcomes on the offspring: depending on the timing of and the adherence to combination antiretroviral therapy (cART), HIV-infected mothers may vertically transmit the virus to their child (HIV-exposed infected, HEI) or give birth to an HIV-exposed uninfected (HEU) baby.

Perinatally HIV-infected children are exposed to both HIV and cART during their life-time, and may be, therefore, at risk of developing noninfectious comorbidities (cardiovascular, renal, bone, neurological) at an extremely early age.

Successfully cART-treated people with HIV (PWH) feature a clinical phenotype of premature aging, the pathogenesis of which is multifactorial. This may be linked, on the one hand, to cART metabolic effects and excess of traditional risk factors [1], and on the other, to the effects of the virus *per se*[2] as well as persistent immune activation/inflammation, which may lead to the increased risk of noninfectious comorbidities [3,4]. As to the latter, gastrointestinal barrier dysfunction is a well established pathogenic mechanism underlying microbial translocation, which in turn associates to immune activation/inflammation. Further, gut dysbiosis also features cART-treated HIV infection [5,6] and correlates with immune activation [7,8] as well as a wide range of noncommunicable disorders [9]. Unsurprisingly, impairment of the gut microbiota features perinatally infected children and adolescents [10–12] and is linked to markers of inflammation [10,11] and vascular endothelial activation [11], highlighting the possible role of a dysbiotic microbiome in driving cardiovascular disease development in this population.

On the other hand, HEU do not experience the effects of HIV infection and cART, yet present a worse clinical outcome compared with their HIV-unexposed uninfected (HUU) counterparts. Indeed, HEU infants display high morbidity and mortality from pneumonia, diarrheal disease and sepsis [13,14] as well as poor growth and neurodevelopment [15]. The underlying reasons for such clinical differences are most likely multifactorial and dependent on the geographical and social setting, maternal health status and breastfeeding [16–19]. Of note, recent data have demonstrated that maternal HIV infection is linked to modifications in the fecal microbiome of HEU with alterations in the abundance of several taxa and functional profiles, suggesting that gut dysbiosis may contribute to the clinical vulnerability in this setting [20,21].

To bridge this knowledge gap, we conducted a pilot study to assess the fecal microbiome, markers of gut damage, microbial translocation and immune activation/inflammation in a cohort of individuals born from HIV-infected mothers and with different HIV serostatus.

## Methods

### Study population

In this cross-sectional, pilot study, we consecutively enrolled individuals born from HIV-infected mothers in active follow-up at one of the participating paediatric centres in Italy (Milan, Modena, Naples, Turin and Brescia). Due to the impact on gut microbiota, children treated with antibiotics or prebiotics/probiotics during the previous 2 weeks were excluded [22,23], as well as children with gastrointestinal disorders or chronic infectious diseases [tuberculosis or hepatitis C virus (HCV)/hepatitis B virus (HBV)/hepatitis D virus (HDV) infection] [24–26]. Individuals on vegan, vegetarian or high-protein diets were also excluded because of the well established impact of such dietary regimens on gut microbiota composition [27,28], as were children younger than 1 year of age because of the physiological immaturity of gut microbiota [29].

On the basis of their HIV serostatus, children were divided into two groups: HEI and HEU.

The study was approved by the local Research Ethics Committees. Written informed consent was obtained from the legal representative of each minor participant.

### Fecal and plasma microbiome analysis

Plasma and fresh stool samples were collected from each individual in EDTA tubes and sterile feces containers, respectively, then immediately frozen and stored until processing at −80 °C.

Total DNA was extracted and amplified in a strictly controlled environment at Vaiomer SAS (Labège, France) using a stringent contamination-aware approach as reported previously [30,31]. The V3–V4 hypervariable regions of the bacterial 16S rDNA, corresponding to 340F-781R positions on the reference *Escherichia coli* sequence, were amplified and quantified by quantitative PCR (qPCR), sequenced with MiSeq technology, and clustered into operational taxonomic units (OTUs) before taxonomic assignment [30].

### Bioinformatic analyses

Targeted metagenomic sequences from microbiota were analyzed using a bioinformatic pipeline established by Vaiomer SAS based on FROGS guidelines [32], as described by Anhê *et al.*[33]. Briefly, the denoising was performed by removing amplicons missing the two PCR primer sequences (10% of mismatches were allowed), amplicons shorter than 350 bases or longer that 480 bases, amplicons with at least one ambiguous nucleotide (’N’), amplicons identified as chimera (with VSEARCH v1.9.5) and amplicons with a strong similarity (coverage and identity ≥80%) with the phiX genome (used as a control for Illumina sequencing runs). Clustering was produced in two passes of the swarm algorithm v2.1.6. The first pass was a clustering with an aggregation distance equal to 1. The second pass was a clustering with an aggregation distance equal to 3. As final denoising step, OTU with very low abundance (≤0.005%) were regarded as sequencing errors, and thus discarded. Taxonomic assignment of amplicons into OTUs was produced by Blast+ v2.2.30+ with the RDP V11.4 database. Reads obtained from the MiSeq sequencing system have been processed using Vaiomer SAS bioinformatics pipeline. The relative proportion taxa for each taxonomic level (phylum, class, order, family, genus and species) were analyzed statistically. Alpha-diversity represents the mean of species diversity per sample in each group/class. Diversity analysis is presented at OUT levels for: richness parameters for species taxa according to (1) observed, (2) Chao1, and (3) PD (Phylogenetic Diversity) indexes; and diversity/evenness parameters for species taxa according to (3) Shannon, (4) Simpson, and (5) inverse Simpson indexes. Principal Coordinate Analysis (PCoA) was performed for comparison of sample groups/class based on four methodologies or beta-diversity: Bray-Curtis (a quantitative measure of community dissimilarity), Jaccard (a qualitative measure of community dissimilarity), Unweighted-Unifrac (a qualitative measure of community dissimilarity that incorporates phylogenetic relationships between the features) and Weighted-Unifrac (a quantitative measure of community dissimilarity that incorporates phylogenetic relationships between the features). Permanova and Permdisp analyses for all beta-diversity indexes were performed. *P* less than 0.05 for both Pseudo *F* and *F* values, respectively, for Permanova and Permdisp, were considered statistically significant. Finally, the output matrix containing the relative abundance of OTUs per sample was processed with the linear discriminant analysis effect size (LEfSe) algorithm [34], using an alpha cut-off of 0.05 for both the factorial Kruskal–Wallis test among classes and the pairwise Wilcoxon test between subclasses, and an effect size cut-off of 2.0 for the logarithmic linear discriminant analysis (LDA) score for discriminative features, and the strategy for multiclass analysis set to ‘all-against-all’.

### Quantification of soluble markers of gut barrier damage, microbial translocation, immune activation and inflammation

Gut barrier function and microbial translocation were assessed through plasma levels of intestinal fatty acid binding protein (I-FABP) (Hycult Biotech, Uden, The Netherlands), E-Cadherin, Endotoxin Core Antibodies (EndoCAb) (R&D Systems, Minneapolis, Minnesota, USA) and soluble CD14 (sCD14). Plasma IL-6 (R&D Systems) was used to evaluate systemic inflammation.

All the biomarkers listed above were measured by ELISA assays following the manufacturers’ instructions.

### Statistical analyses

Continuous variables were expressed as medians and interquartile ranges (IQR), whereas categorical variables as absolute numbers and percentages. Data in the two groups were compared by chi-squared/Fisher's exact and Mann–Whitney *U* tests wherever appropriate by GraphPad Prism 6.2 (GraphPad Software Inc., San Diego, California, USA).

## Results

### Study population

Eighty participants were enrolled. On the basis of their HIV serostatus, 47 were HEI and 33 HEU; the latter all received antiretroviral therapy and, with the exception of three participants, had undetectable viral load at the time of the study. The median age was 14 and 12 years for HEI and HEU children, respectively. Thirty-six of 80 (45%) children were girls (HEI: *n* = 19, HEU: *n* = 17) and 21 of 80 patients (26%) were born in African countries (HEI: *n* = 16, HEU: *n* = 5).

As expected, the two groups differed significantly in the type of feeding at birth, mode of delivery, mother's cART during pregnancy and postexposure prophylaxis. In detail, 22 of 80 patients were breastfed (HEI: *n* = 21, HEU: *n* = 1; *P* < 0.0001), 44 of 80 children were born by caesarean section (HEI: *n* = 18, HEU: *n* = 26; *P* = 0.0005) and 26 of 80 by vaginal delivery; in 10 of 80 children, the mode of delivery was unknown. Of the 34 children born from mothers who received standard triple cART during pregnancy, five were HEI and 29 HEU (*P* < 0.0001). Postexposure prophylaxis with zidovudine was administered to 13 (28%) HEI and 26 (79%) HEU (*P* < 0.0001).

No significant statistical difference was observed between the two groups in terms of birth weight and preterm birth occurrence.

Demographic and epidemiological characteristics of study participants are presented in Table 1. Viro-immunological characteristics of HEI are summarized in Table 2.

**Table 1:** Demographic and epidemiological characteristics of study patients.

Characteristic	Total (*n* = 80)	HEI (*n* = 47)	HEU (*n* = 33)	*P* value (HEI vs. HEU)
Sex, female [*n*, (%)]	36 (45)	19 (40)	17 (51)	0.367
Country of birth [*n* (%)]				0.073
Africa	21 (26)	16 (34)	5 (15)	
Europe	59 (73)	31 (66)	28 (85)	
Age (years) [median (IQR)]	13 (8–16)	14 (9–16)	12 (4.5–14.5)	0.062
Mode of delivery [*n* (%)]				0.0005
Vaginal	26 (33)	23 (49)	3 (9)	
Caesarean section	44 (55)	18 (38)	26 (79)	
Unknown	10 (12)	6 (13)	4 (12)	
Weight at birth (g) [median (IQR)]	3000 (2702–3295)	3000 (2705–3240)	3100 (2637–3340)	0.523
Breastfed [*n* (%)]	22 (27)	21 (45)	1 (3)	<0.0001
Preterm birth [*n* (%)]	12 (15)	7 (15)	5 (15)	1
BMI SD [*n* (%)]				
Normal (−1.86 < BMI SD < +1.86)	53 (66)	39 (83)	14 (42)	0.0001
Low (BMI SD < −1.86)	0	0	0	
High (BMI SD > +1.86)	12 (15)	6 (13)	6 (18)	
Unknown	15 (19)	2 (4)	13 (40)	
Mothers on standard triple cART during pregnancy [*n* (%)]	34 (43)	5 (11)	29 (88)	<0.0001
Infant postexposure prophylaxis [*n* (%)]	39 (49)	13 (28)	26 (79)	<0.0001

Postexposure prophylaxis was administered with zidovudine (2 mg/kg, four times daily). cART, combination antiretroviral therapy; HEI, HIV-exposed infected; HEU, HIV-exposed uninfected; IQR, interquartile range; preterm birth, birth occurring before 37 completed weeks of gestation; BMI SD, BMI standard deviation (corrected for age and sex).

**Table 2:** Viroimmunological characteristics of HIV-exposed infected.

Characteristic	HEI (*n* = 47)
CD4^+^ T-cell count [median (IQR)]	
cells/μl	728 (532–945)
%	34 (30–39)
CD8^+^ T-cell count [median (IQR)]	
cells/μl	621 (461–892)
%	32 (25–39)
CD4^+^/CD8^+^ ratio [median (IQR)]	1.12 (0.79–1.32)
Detectable viral load [*n* (%)]	3 (6)
On cART [*n* (%)]	47 (100)
cART regimen at the time of study [*n* (%)]	
NNRTI	4 (9)
PI	20 (42)
INSTI	23 (49)
Time on current cART (months) [median (IQR)]	15 (9–35)

cART, combination antiretroviral therapy; HEI, HIV-exposed infected; INSTI, integrase strand transfer inhibitor. IQR, interquartile range; NNRTI, nonnucleoside reverse transcriptase inhibitor; PI, protease inhibitor.

### HIV-exposed infected and HIV-exposed uninfected individuals display different fecal beta-diversity and relative abundance, yet similar microbiome profiles in plasma

**Fig. 1:**
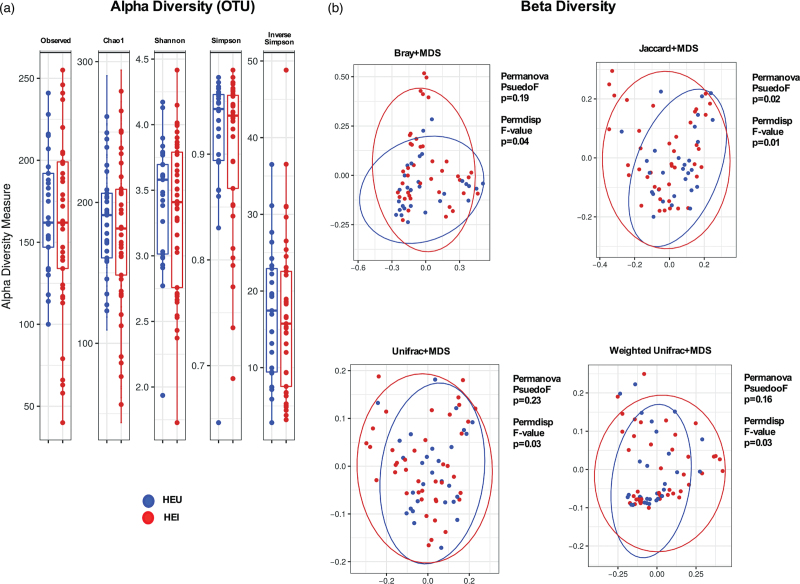
Alpha and beta-diversity parameters of the fecal microbiome in HIV-exposed infected and HIV-exposed uninfected.

**Fig. 2:**
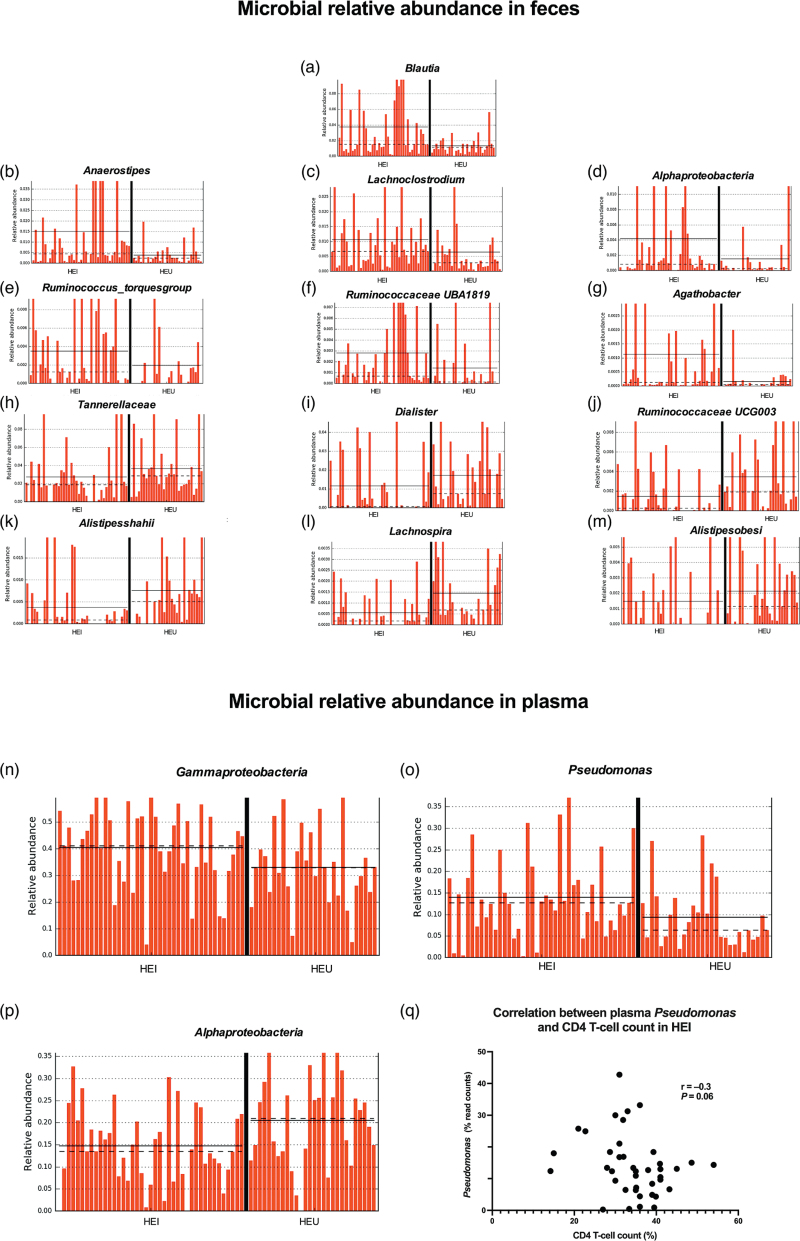
Microbial relative abundance in fecal and plasma samples from HIV-exposed infected and HIV-exposed uninfected and correlation with CD4^+^ T-cell counts.

We first analyzed the fecal microbiome in HEI and HEU. The two groups displayed similar taxonomic composition at the phylum, class, order, family, genus and species levels in fecal samples (Supplementary Figure 1). No differences were observed in alpha-diversity richness and evenness indexes (Fig. 1a). Beta-diversity measures (Bray, Jaccard, Unifrac and Weighted Unifrac) between groups were significantly different upon Permdisp (respectively, *P* = 0.04, *P* = 0.01, *P* = 0.03, *P* = 0.03), yet not Permanova analysis (Fig. 1b). LDA effect size (LEfSe) showed differences in relative abundance with outgrowth of *Blautia* spp., *Anaerostipes* spp., *Lachnoclostridium* spp., Alphaproteobacteria, *Ruminococcus torques* group, Ruminococcaceae UBA1819 and *Agathobacter* spp. in HEI (Fig. 2a–g) and Tannerellaceae, *Dialister* spp., *Alistipes shahii*, Ruminococcaceae UCG003, *Lachnospira* spp., *Alistipes obesi* in HEU (Fig. 2h–m).

(a) Analysis of alpha-diversity (OTU level) by Kruskal–Wallis resulted in nonsignificant differences between HEI and HEU: observed (*P* = 0.70), Chao1 (*P* = 0.67), Shannon (*P* = 0.49), Simpson (*P* = 0.47), inverse Simpson (*P* = 0.48). (b) Analysis of beta-diversity showed a significant separation between HEI and HEU upon Permdisp, yet not Permanova test of the following indexes: Bray, Jaccard, Unifrac, Weigthed Unifrac. Post hoc tests were calculated when the global test's *P* value was below 0.05. Post hoc *P* values are corrected using the Benjamini–Hochberg method. HEI, HIV-exposed infected; HEU, HIV-exposed uninfected; OUT, operational taxonomic unit.

Linear discriminant analysis (LDA) effect size (LEfSe) with LDA score greater than 2 as the cut-off showed differences in relative abundance between groups. HEI, HIV-exposed infected; HEU, HIV-exposed uninfected.

Plasma microbiome analysis in HEI and HEU did neither show significant differences in alpha-diversity (Observed, *P* = 0.84; Chao1, *P* = 0.84; Shannon, *P* = 0.16; Simpson, *P* = 0.26; InvSimpson, *P* = 0.25) nor beta-diversity measures (Permanova pseudo *F* and Permdisp *F* value, respectively: Bray, *P* = 0.12 and *P* = 0.64; Unifrac, *P* = 0.10 and *P* = 0.45; weighted Unifrac, *P* = 0.15 and *P* = 0.88; Jaccard, *P* = 0.08 and *P* = 0.47). Of note, the study of relative abundance in plasma showed a different profile than that of feces, with a predominance of Gammaproteobacteria and *Pseudomonas* spp. in HEI (Fig. 2n–o) and of Alphaproteobacteria in HEU (Fig. 2p).

Interestingly, although no correlations were found between fecal microbiota composition and immune reconstitution, a trend towards a negative correlation between plasma *Pseudomonas* spp. and CD4^+^ T-cell percentages was found in HEI (*r* = −0.3, *P* = 0.06; Fig. 2q).

### Higher monocyte activation and inflammation in HIV-exposed infected than HIV-exposed uninfected, despite comparable levels of gut damage and microbial translocation in the two groups

**Fig. 3:**
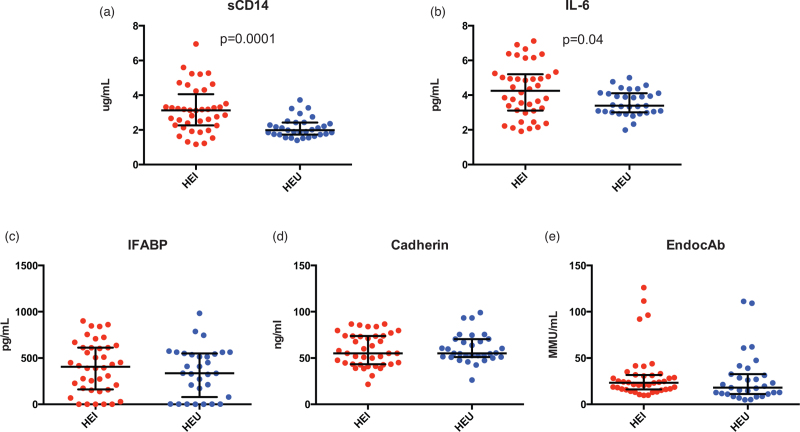
Inflammation, gut damage and microbial translocation in HIV-exposed infected and HIV-exposed uninfected.

EndoCAb, endotoxin core antibodies; IFABP, intestinal fatty acid-binding protein; IL-6, interleukin-6; sCD14, soluble CD14^+^.

We next measured markers of immune activation/inflammation as well as gut damage and microbial translocation in HEI and HEU. As expected, HEI presented significantly higher sCD14 [3.1 (2.3–4.1) vs. 2.0 (1.7–2.4) μg/ml; *P* = 0.0001; Fig. 3a] and IL-6 levels [4.3 (3.1–5.2) vs. 3.4 (3.1–4.1) μg/ml; *P* = 0.04; Fig. 3b] than HEU. In contrast; however, similar levels of peripheral IFAB-P [405.5 (160.8–611.4) vs. 335.2 (78.1–549.4) pg/ml; *P* = 0.5; Fig. 3c], E-cadherin [55.0 (43.3–73.9) vs. 55.0 (51.0–70.4) ng/ml; *P* = 0.6; Fig. 3d] and EndoCAb [23.3 (16.1–31.7) vs. 17.9 (11.1–32.5) mMU/ml; *P* = 0.2; Fig. 3e] were found in HEI and HEU, respectively.

No correlations were found between the fecal microbiome and gut function/microbial translocation markers in HEI.

## Discussion

In a cohort of individuals born from HIV-infected mothers, the present study shows that patients who acquire infection (HEI) display a different fecal microbiome and higher systemic inflammation than those who remain HIV-uninfected (HEU), despite comparable gut barrier function, microbial translocation and plasma microbiome.

In particular, individuals exposed to HIV *in utero* present similar taxonomic composition and alpha diversity in feces, yet harbor diverse microbiomes in terms of relative abundance according to the outcome of maternal infection: indeed, while HEI display an outgrowth of *Blautia* spp., *Anaerostipes* spp., *Lachnoclostridium* spp., Alphaproteobacteria, *R. torques* group, Ruminococcaceae UBA1819, and *Agathobacter* spp., HEU present higher Tannerellaceae, *Dialister* spp., *A. shahii*, Ruminococcaceae UCG003, *Lachnospira* spp. and *A. obesi*. These results, together with our findings of increased sCD14 and IL-6 in HEI, are in partial contrast with data from another study showing that high fecal biodiversity with enrichment of Blautia and *R. torques* in individuals with perinatal infection associate with lower biomarkers of inflammation, monocyte activation and vascular endothelial activation [11]. Indeed, we failed to describe a correlation between inflammatory markers and the gut microbiome in HEI. The fecal microbial signature that we report in HEI has been shown to exert anti-inflammatory properties in the gut [35,36], which may explain the comparable levels of I-FABP, cadherin and EndocAb in HEI and HEU. In keeping with this observation, our results of increased Ruminococcaceae UCG003 and *Lachnospira* spp. in HEU are in accordance with prior research [21] indicating, in this population, a possible maintenance of the gut epithelial barrier through the production of short-chain fatty acids by Lachnospiraceae [37].

The above-mentioned differences in the fecal microbiome were observed through significant Permdisp but not Permanova analysis of beta-diversity parameters. Such a discrepancy suggests that the difference between the two study groups relies more on within-group distances (dispersion) than between-group distances (location). In other words, fecal microbiota of HEI as a whole is not strikingly different to that of HEU overall; however, although HEU individuals have a more similar fecal microbiota to each other, HEI exhibit a higher variability in fecal microbiota among themselves, possibly mirroring nuances of perinatal HIV exposure in shaping fecal microbioma beyond infancy.

In contrast to fecal microbiome findings, HEI and HEU showed similar microbiome signatures in plasma with only slight differences in relative abundance. In this respect, we found a weak negative correlation between plasma *Pseudomonas* spp. and CD4^+^ T-cell counts in HEI, whereas, in disagreement with others [38–41], the fecal microbiome did not associate with immune reconstitution.

Aside from a possible beneficial role of the fecal microbiome on the intestinal mucosa, our findings of comparable microbial translocation and gut function markers in HEI and HEU may also point to cART-mediated improvement of gut barrier alterations, which feature untreated HIV infection. In fact, a prior study demonstrated higher I-FABP levels in untreated HEI compared with HEU, thus suggesting that gut barrier damage might feature perinatal HIV infection but not necessarily intrauterine HIV exposure [42]. Given that HEI enrolled in our research are all on treatment, we speculate that suppressive cART may, at least in part, be accountable for our findings of similar I-FABP, cadherin and EndocAb in HEI and HEU.

As opposed to the above, sCD14 and IL-6 were significantly higher in HEI compared with HEU and confirm how immune activation and inflammation are specific to HIV-infected individuals albeit effective cART [2,42]. Together with our reported findings of similar gut structure/function, microbial translocation and composition of the plasma microbiome in HEI and HEU, these data suggest that, in the setting of perinatally acquired HIV infection, residual immune activation and inflammation on effective cART are seemingly not linked to alterations within the intestinal mucosa. However, they cannot rule out that the observed modifications of the gut microbiota in HEI may have a role in fueling persistent peripheral immune activation and inflammation [10].

Some limitations to microbiome data interpretation should be acknowledged, including its cross-sectional/pilot design, which does not allow to adjust for mode of delivery and breastfeeding, factors known to shape the composition of the microbiota [19,43–48]. Likewise, the lack of a HUU control group limits the possibility to weigh the effects of intrauterine viral exposure vs. vertical HIV infection.

Furthermore, although our approach of V3–V4 hypervariable region may not provide enough taxonomic resolution to accurately assess differences at the species level, within some bacterial genera, the sequence differences in-between species that we detected were large enough to allow species-level taxonomic assignation from highly significant alignments.

Despite its limitations, our study expands the bulk of existing knowledge on how maternal HIV infection differently impacts the composition of the offspring gut microbiota according to HIV serostatus. Indeed, the vast majority of research performed until now focused on the comparison between HIV-exposed (HEI/HEU) and unexposed (HUU) children, therefore failing to dissect potential dissimilarities in gut microbiota, gut barrier function, immune activation and pro-inflammatory status in the two distinct populations born form HIV-infected mothers.

Future studies on HEI, HEU and HUU controlling for potential confounders are needed to understand the role of gut-mediated alterations in the clinical risk of perinatally HIV-exposed individuals beyond infancy and inform on the best clinical strategies to manage and mitigate excess morbidity in this population.

## Acknowledgements

We are thankful to all the patients who participated in the study and the staff of the Clinic of Infectious Diseases and Tropical Medicine, San Paolo Hospital, ASST Santi Paolo e Carlo, Department of Health Sciences, University of Milan who cared for them.

Funding: the study was supported by i) the Italian Ministry of Health, Regione Lombardia, grant ‘Giovani Ricercatori’ (number GR-2009-1592029) and grant ‘Ricerca Finalizzata’ (number NET-2013-02355333-3), both awarded to G.M. ii) ERANET-Transcan 2, Third Joint Transnational Call (JTC, 2016), funding agency: Fondazione Regionale della Ricerca Biomedica (FRRB; grant ID 073) awarded to C.T.

Author contributions: C.Ti. participated in the study conceptualization, elaborated and analyzed clinical and laboratory data and wrote the paper; M.F. conceptualized the study, recruited patients, collected clinical data, analyzed clinical data and participated in article writing; F.F. collected and analyzed patients clinical data and edited the article; M.A. participated in data analyses and article writing; L.D. recruited patients, collected clinical data and edited the manuscript; C.Ta. recruited patients, collected clinical data and edited the manuscript; A.D. recruited patients, collected clinical data and edited the manuscript; V.C. recruited patients, collected clinical data and edited the manuscript; L.I. participated in the study conceptualization and article editing; R.B. participated in the study conceptualization and article editing; M.C. conceptualized the article, analyzed clinical data and participated in article writing; G.M. conceptualized the article, analyzed clinical and laboratory data and wrote the manuscript.

**MIRROR Study Group**: author contributors – Eugenia Bruzzese, Manuela Cortesi, Antonella d’Arminio Monforte, Giada Di Pietro, Clara Gabiano, Silvia Garazzino, Ilaria Mariotti, Alessia Pancaldi.

### Conflicts of interest

There are no conflicts of interest.

This work was presented in part at the 18th European AIDS Conference (EACS), London, UK, 2021 (poster #BPD4/6).

## Supplementary Material

Supplemental Digital Content
