# Supplementary figures and images for: Gut-dependent inflammation and alterations of the intestinal microbiota in individuals with perinatal HIV exposure and different HIV serostatus
Source: AIDS. 2022 Jul 22;36(14):1917–25. doi: 10.1097/QAD.0000000000003324 (PMC9612678; doi:10.1097/QAD.0000000000003324)

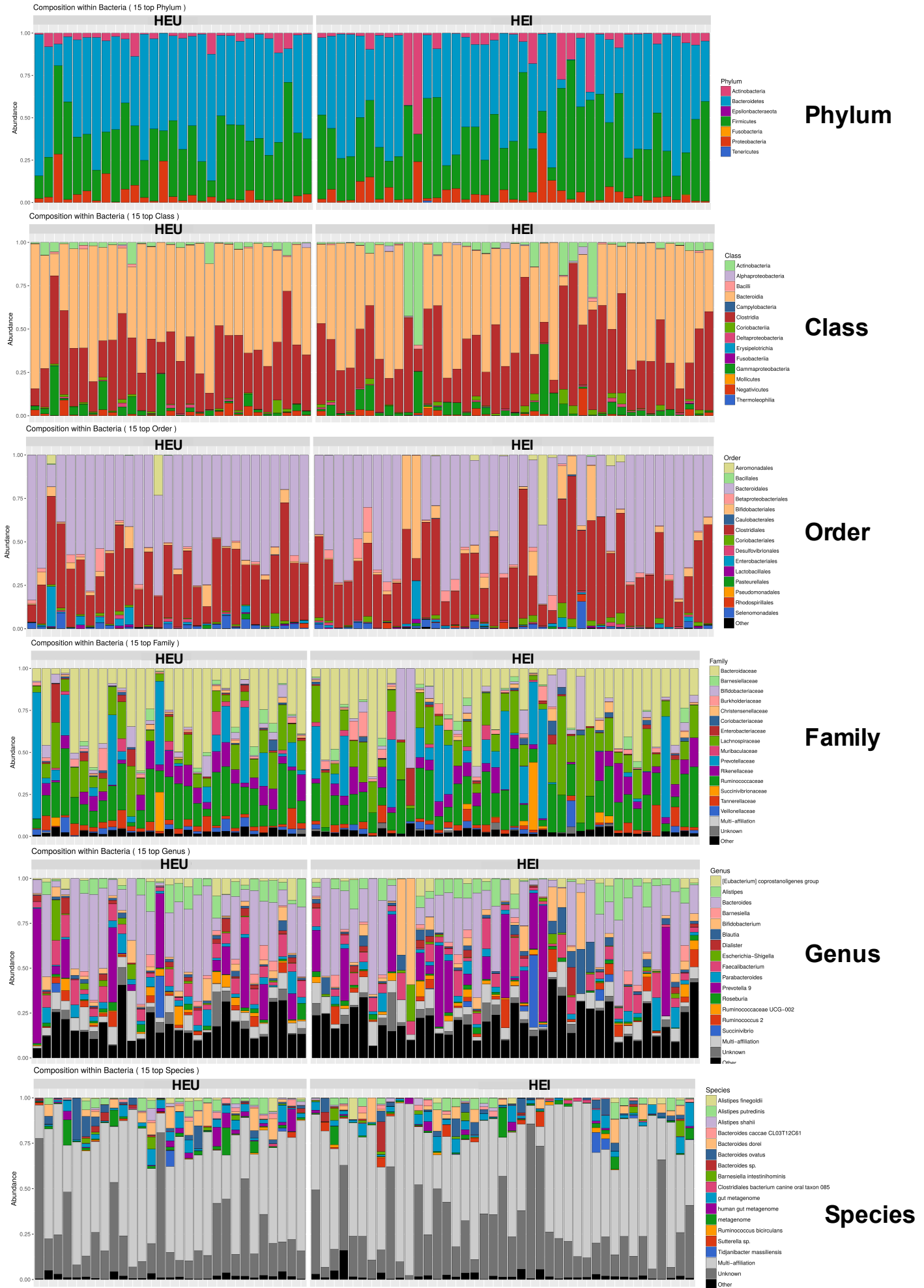

Supplement: Supplemental Digital Content [file aids-36-1917-s001.pdf]
